# Modelling the Renal Excretion of the Mycotoxin Deoxynivalenol in Humans in an Everyday Situation

**DOI:** 10.3390/toxins13100675

**Published:** 2021-09-22

**Authors:** Annick D. van den Brand, Rudolf Hoogenveen, Marcel J. B. Mengelers, Marco Zeilmaker, Gunnar S. Eriksen, Silvio Uhlig, Anne Lise Brantsæter, Hubert A. A. M. Dirven, Trine Husøy

**Affiliations:** 1 National Institute for Public Health and the Environment (RIVM), 3721 MA Bilthoven, The Netherlands; rudolf.hoogenveen@rivm.nl (R.H.); marcel.mengelers@rivm.nl (M.J.B.M.); marco.zeilmaker@rivm.nl (M.Z.); 2 Norwegian Veterinary Institute (NVI), P.O. Box 64, 1431 Ås, Norway; gunnar.eriksen@vetinst.no (G.S.E.); silvio.uhlig@vetinst.no (S.U.); 3 Norwegian Institute of Public Health (NIPH), N-0213 Oslo, Norway; annelise.brantsaeter@fhi.no (A.L.B.); Hubert.Dirven@fhi.no (H.A.A.M.D.); trine.husoy@fhi.no (T.H.)

**Keywords:** deoxynivalenol, deoxynivalenol-15-glucuronide, mycotoxin, human biomonitoring, dietary exposure, renal excretion, human study

## Abstract

The dietary exposure to the mycotoxin deoxynivalenol (DON) can be assessed by human biomonitoring (HBM). Here, we assessed the relation between dietary DON intake and the excretion of its major metabolite DON-15-glucuronide (DON15GlcA) through time, in an everyday situation. For 49 volunteers from the EuroMix biomonitoring study, the intake of DON from each meal was calculated and the excretion of DON and its metabolites was analyzed for each urine void collected separately throughout a 24-h period. The relation between DON and DON15GlcA was analyzed with a statistical model to assess the residence time and the excreted fraction of ingested DON as DON15GlcA (f_abs_excr_). F_abs_excr_ was treated as a random effect variable to address its heterogeneity in the population. The estimated time in which 97.5% of the ingested DON was excreted as DON15GlcA was 12.1 h, the elimination half-life was 4.0 h. Based on the estimated f_abs_excr_, the mean reversed dosimetry factor (RDF) of DON15GlcA was 2.28. This RDF can be used to calculate the amount of total DON intake in an everyday situation, based on the excreted amount of DON15GlcA. We show that urine samples collected over 24 h are the optimal design to study DON exposure by HBM.

## 1. Introduction

Deoxynivalenol (DON) is a foodborne mycotoxin that is produced by several *Fusarium* spp. These fungi can infect various types of grains in the field and thus, DON is commonly found in grains after harvest and subsequently in grain-based products. The European Food Safety Authority (EFSA) estimated in 2017 that the European adult population is chronically exposed to DON, sometimes around the level of the established tolerable daily intake (TDI) [1]. The level of mycotoxin contamination in food depends on various factors such as *Fusarium* strain, crop species, temperature, and humidity during crop growth. A recently published report on climate change as a driver of emerging risks for food and feed safety indicated that the exposure to DON is expected to increase as a result of climate change [2].

Moreover, other forms of DON have also been reported, such as the plant metabolite of DON, DON-3-glucoside (DON3G), which is called a modified mycotoxin. DON3G is almost completely converted to DON in the human body upon digestion and, therefore, DON3G contributes to the total exposure of DON [3,4]. Other forms of DON that have been reported to occur in foods are 3-acetyl-DON (3ADON) and 15-acetyl-DON (15ADON), and to a minor extent, modified DON glutathione-conjugates [1,5,6]. 

Consumption of heavily DON-contaminated food can result in acute DON-induced effects on the GI tract of animals and humans, including vomiting (hence DON’s trivial name vomitoxin). The chronic exposure of low amounts of DON can result in effects such as immune and developmental effects and reduced body weight gain, which have been reported in animal studies. EFSA derived a group TDI for DON, DON3G, 3ADON, and 15ADON of 1 µg per kg body weight per day (µg/kg bw/day) [1]. This value was also derived by the Joint FAO/WHO Expert Committee on Food Additives (JECFA) as a Provisional Maximum Tolerable Daily Intake for DON and its acetylated derivatives [7]. DON3G is not included in this JECFA reference value as, at that time, it was not yet reported that DON3G is converted to DON in the GI tract in humans. To protect consumers, the European Commission has set maximum limits for DON in several food commodities [8].

Traditionally, the exposure to chemicals in a population is assessed by linking consumption data of individuals to occurrence data (from monitoring programs). EFSA calculated in 2017 that the exposure to DON varied considerably throughout Europe, with some populations exceeding the TDI in the 95th percentile of the exposure distribution of adolescents and adults, and even at the 50th percentile for infants, toddlers, and children [1]. 

Another approach to assess the exposure to chemicals is via human biomonitoring (HBM). HBM is a tool to assess the exposure of a population to compounds or their metabolites, in human matrices such as blood or urine. Whenever this internal exposure is used to calculate the external exposure (by means of modelling) this is called “backward modelling of exposure”. The advantage of backward exposure modelling is that it can show what the actual exposure of an individual was at a specific point in time. In addition, the exposure to modified or acetylated forms of DON, which are converted to DON in the human body, are also taken into account with HBM. This is an advantage, since these (modified) forms are often not (yet) routinely analyzed in food monitoring, which will result in an underestimation of exposure. On the other hand, traditional exposure assessments can be used to identify the major contributing source(s) of exposure, which is often not possible with HBM. Therefore, HBM and traditional exposure assessments greatly complement each other in risk assessment. 

Urine is a good HBM matrix for DON, as DON is mainly excreted in the urine (as metabolites) accounting for approximately 70% of the total intake [3,9,10,11,12,13], and urine samples are easily collected in cohort studies. The main metabolic pathway in DON elimination is via glucuronidation, with deoxynivalenol-15-glucuronide (DON15GlcA) being the major metabolite [3,4,14,15,16]. DON is excreted relatively rapidly: within 12 h, 95% of the total elimination products are excreted via urine [3,9]. Like DON, DON3G is excreted via urine as well [9].

Previously, the excretion of DON and DON3G was studied in a human intervention study based on a single-dose of DON and DON3G at the level of the TDI [3]. The excretion of both DON and DON3G, and their metabolites, was followed up to 24 h after ingestion. Based on this information a biokinetic model was developed by our group [9].

In this study, we assessed the relation between DON intake and DON (metabolites) excretion through time, in an everyday situation rather than an experimental setting. To this end, the total DON intake from each meal throughout a 24-h period was estimated using detailed information on the individual consumption and DON occurrence data. All urine voids during that period were collected separately and analyzed for DON and its metabolites for a total of 49 participants from the EuroMix biomonitoring study [17]. A statistical model was developed by fitting the optimal model parameters using both intake and excretion data of DON. In future studies, this model can be used to assess the external exposure to DON in a population, based on urinary data.

## 2. Results

The individual intake and patterns of dietary DON consumption were examined, as well as the excretion of DON and its metabolites in urine voids collected separately throughout a 24-h period. Of the analyzed urinary DON and metabolite concentrations, only DON’s major metabolite DON15GlcA was analyzed above the limit of detection for a sufficient number of urine voids per participant. Therefore, the relation between DON intake and excretion could only be estimated for DON15GlcA (see Materials and Methods). To that end, a mathematical mixed effects regression model was developed that describes the excreted fraction (f_abs_excr_) and the time from DON intake to DON15GlcA excretion in urine (residence time). The residence time was described using various statistical distributions, namely the gamma distribution, the log-normal distribution, and the exponential distribution, in order to account for the uncertainties related to distribution models. The gamma distribution provided the best fit for the residence time, as indicated by the lowest Akaike Information Criteria (AIC).

### 2.1. Dietary Intake of DON

We estimated the dietary intake of total DON of all participants throughout a 24-h period using detailed information on the individual consumption and DON occurrence data (see Materials and Methods). The estimated median dietary intake of the 49 participants varied between 0.43–0.75 µg/kg bw/day, depending on the estimated variation of DON in food items, and the estimated 95th percentile of intake varied between 0.77–1.38 µg/kg bw/day. The minimum estimated daily dietary exposure varied between 0.13–0.24 µg/kg bw/day and the maximum exposure between 0.91–1.65 µg/kg bw/day. The dietary intake of DON3G was also accounted for, by multiplying the DON intake with an estimated factor for DON3G co-occurrence, as expressed on a molar basis (see Material and Methods). As the selected participants in this study are not a representative sample of the Norwegian population, we do not consider this a risk assessment of DON in Norway (see Materials and Methods).

**Figure 1:**
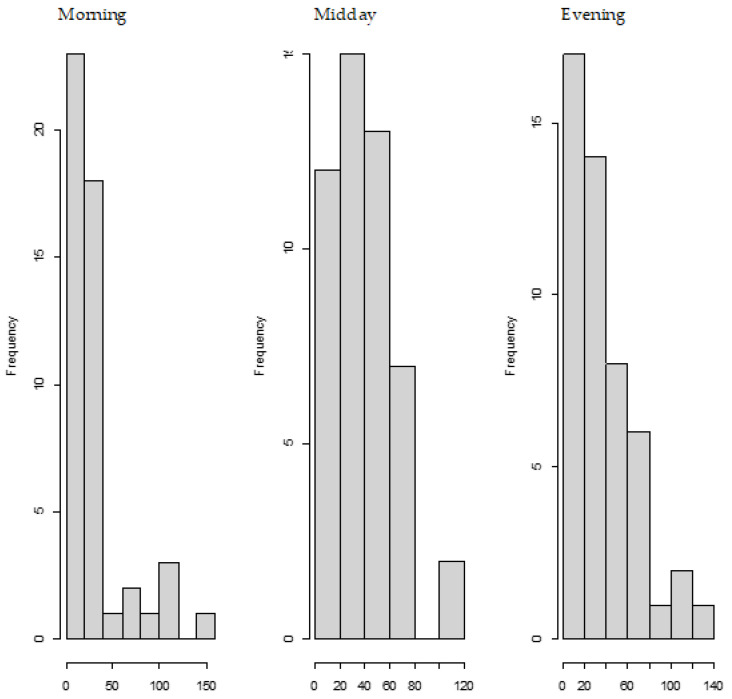
Frequency of consumed amounts of DON during different parts of the day. The amounts of DON in nanomoles per consumption moment are expressed on the x-axis and the frequency of those consumed amounts are expressed on the y-axis. Different parts of the day are divided in morning (<10:00), midday (between 10:00 and 16:00) and evening (>16:00).

Figure 1, shows the frequency of the consumed amounts of DON during three parts of the day (<10:00, 10:00–16:00 and >16:00). The lowest amounts of DON per intake moment in Norway are consumed most often in the morning, and the highest during the latest part of the day (>16:00). We also observed that the average (standard deviation) total DON intake consumed during these three parts of the day were subsequently 27% (21%), 40% (26%) and 33% (26%) as compared to the total intake. However, a very large variation in those fractions was observed, which explains that although the highest amounts of DON are consumed during the latest part of the day, the average intake is higher during midday.

**Figure 2:**
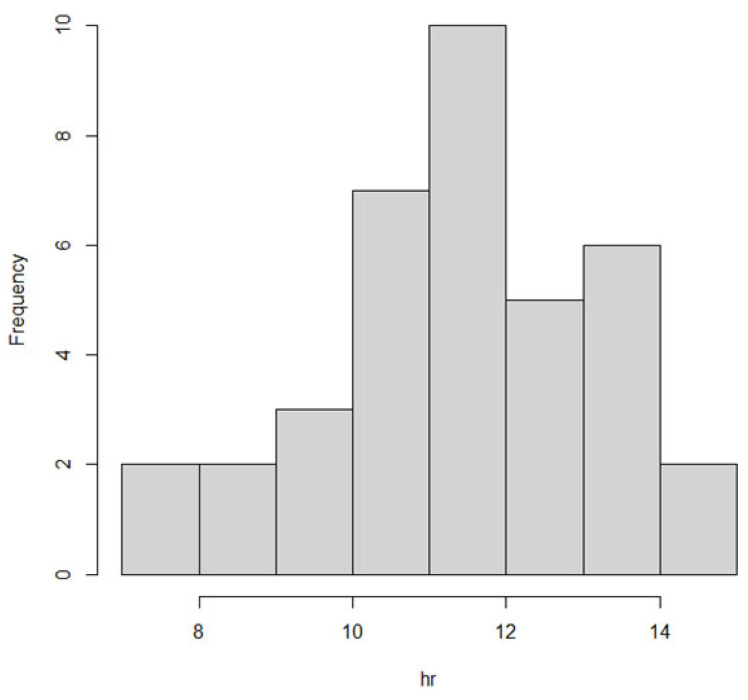
Distribution of the weighted time between the estimated DON intake after 16:00 h and the morning urine void the next day. The time between the intake and excretion is plotted against the frequency of that time observed in the individuals.

In addition, we analyzed the weighted time between the evening intake of DON and the morning urine the following day, in order to assess to what extent the DON in the evening meal is excreted in the following morning urine void. Figure 2 shows that the median time between DON intake after 16:00 h and before the morning urine the next day is 11.5 h, with a minimum time of 7.1 h and a maximum time of 14.8 h.

### 2.2. Urinary Excretion of DON

The total amount of DON15GlcA excreted throughout the 24-h period was on average 26.1 µg. This amount was higher in males (average 33.4 µg) as compared to females (average 18.6 µg) (Table 1). The urine voids in which DON15GlcA was analyzed below the limit of detection, were assumed to contain zero DON15GlcA (see Materials and Methods). In addition, the total volume of the urine voids over 24 h was on average 2107 mL, or 29.1 mL/kg bw. This also differed between males (26.6 mL/kg bw) and females (31.8 mL/kg bw).

### 2.3. Model

#### 2.3.1. Residence Time

Table 2 shows the mean, the standard deviation, the median (P50), and the 97.5th percentile of the distribution of the estimated residence time in the model using the various statistical distributions. The median of the estimated residence time (i.e., half-life of DON15GlcA elimination) was 3.97 h, 3.58 h, and 2.00 h using the gamma, the log-normal, and the exponential distribution, respectively. The 97.5th percentile of the residence time was 12.1 h, 10.4 h, and 10.7 h using the gamma, log-normal, and the exponential distribution, respectively. The preferred statistical distribution to describe the excretion of DON15GlcA over time is the gamma distribution based on the lowest Akaike Information Criterium (AIC) value (see Materials and Methods). The individual analyzed data and fitted data of the 39 individuals included in the modelling, is shown in Appendix G (see Materials and Methods).

#### 2.3.2. Reversed Dosimetry Factor

The distribution of the excreted fraction of DON as DON15GlcA (f_abs_excr_) was also assessed using the mathematical model and is shown in Table 2. The mean f_abs_excr_ was 0.44 using the gamma distribution for the residence time, 0.47 using the log-normal distribution, and 0.44 using the exponential distribution. Based on the f_abs_excr_ and the identified relative uncertainty in the model, we estimated a reversed dosimetry factor (RDF) for the population mean (with 95% confidence interval) of 2.28 (1.88–2.76), 2.14 (1.74–2.64), and 2.27 (1.84–2.80) using the gamma distribution, the log-normal distribution, and the exponential distribution for the residence time, respectively. The distribution of the individual RDF values (reflecting heterogeneity in the population) ranges from 0.88–5.91 (centered around the population mean value of 2.28) using the gamma distribution. The variation between the individuals was relatively high, compared to the uncertainty of the mean value.

#### 2.3.3. Visualization of Excretion

**Figure 3:**
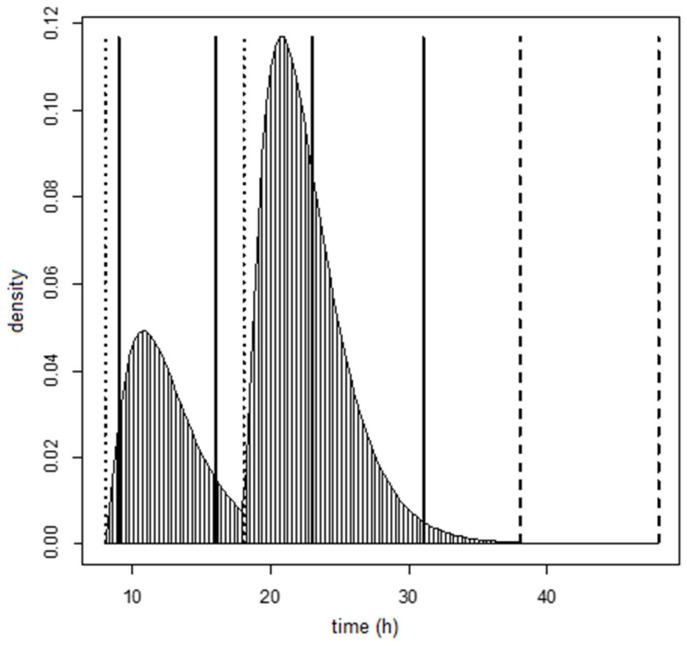
The density function of the statistical model for the excretion of DON15GlcA in a fictional individual, assuming the residence time parameters of the statistical model using the gamma distribution for the residence time. The amount of DON15GlcA excreted in urine is reflected by the area under the curve between two excretion time points (vertical black lines at t = 9, 16, 23, and 31 h). The modeled additional excreted amounts of DON15GlcA are generated at t = 38 and t = 48 h (dashed lines). Intake time points were assumed at t = 8 and t = 18 h (dotted lines). Daily time in hours is expressed at the x-axis. Note that the choice for the residence time is independent from the design of the model, it only affects the visualization.

The design of the statistical model and consequent excretion of DON15GlcA is visualized for a fictional individual in Figure 3, using the gamma distribution for the residence time (note that the choice for the residence time is independent from the design of the model, it only affects the visualization). The design contains the set of known data points (within the 24-h window) and unknown generated data points (after the 24-h window) that were used in the analyses. The curve shows the probability density function of the DON15GlcA excretion based on two hypothetical DON intake values, a fraction of the total intake of 0.3 at 8:00 (t = 8 h), and a fraction 0.7 at 18:00 (t = 18 h) (shown as dotted lines), and four excretion time points, at 9:00, 16:00, 23:00, and 7:00 the following day (t = 9, 16, 23, and 31 h, shown as vertical lines). The additional unknown excretion time points during the following day are generated at t = 38 and 48 (shown as vertical dashed lines). The shaded area between t = 31 and t = 38 is the missing (non-zero) excreted amount at t = 38. The area between t = 38 and t = 48 is the excreted amount at t = 48, which is assumed to be zero-valued. How we dealt with missing values relating to unknown intake data before the 24-h window can be found in Appendix D. The total area under the curve between the excretion time points represents the measured cumulative excreted amount in urine between those time points. Since the fraction of DON that is excreted as DON15GlcA varies between individuals, we assumed that this fraction was 1 in this fictional individual. Therefore, the area under this curve will be 1 (0.3 + 0.7). Note that there is a small fraction of DON15GlcA that is excreted in urine after the 24-h study period (at t = 31 h), when the last DON intake is at t = 18 (see Materials and Methods).

## 3. Discussion

In this study, we modelled the distribution of the DON15GlcA residence time and excreted fraction following DON intake in an everyday situation. This was done using the estimated dietary intake data of DON and the excreted DON15GlcA of separate urine voids throughout a 24-h period of each individual participant. We developed this model so that it can be used in future studies to assess the external exposure to DON in a population, based on only urinary data.

The dietary intake of DON was estimated using the lowest and highest mean occurrence values of DON concentrations measured in four different Norwegian flour products during years 2008 to 2011. The DON analyses were conducted by the Norwegian Veterinary Institute, which is the National Reference Laboratory for mycotoxins in food and feed, and the results comprise the most recent national monitoring data for mycotoxins in food. The Norwegian occurrence data were complemented by the mean medium-bound DON concentrations from the European occurrence data for pasta, rye bread, and breakfast cereals other than oats, reported in the EFSA risk assessment of DON [1], see Materials and Methods. Estimating dietary intake of DON based on self-reported food consumption is associated with uncertainties, both in food intake and in the occurrence of DON in food. Although weighted food records, as used in this study, are considered to provide more precise estimates of consumed portions than retrospective recall, participants may misreport their food intake. The distribution of DON in food is heterogeneous, while we have applied the same DON value for identical food products. This deterministic approach implies that every slice of, e.g., white bread would contain the same amount of DON. At an individual basis, this is unlikely in reality, as we estimated a dietary intake of DON for a few individuals based on their reported consumption but did not observe DON15GlcA in their urine. This discrepancy may, however, also be related to diluted urine voids, resulting in DON15GlcA concentrations below the level of detection. Indeed, relatively high urine volumes were collected from the three participants for which no DON15GlcA was analyzed in urine. It should also be noted that the calculated DON intake was also based on the highest mean occurrence values in Norwegian flour, which should be considered a “worst case scenario” and is not a realistic estimate of the chronic DON intake on its own [18]. These occurrence values were considered to account for the annual variation in DON occurrence. Yet, good correlations between urinary DON morning urine samples and DON intake based on the lowest mean occurrence values in Norwegian flour products were found [19].

In addition, we accounted for the dietary exposure of DON3G, as DON often co-occurs with other forms of DON. Since the modified and acetylated forms of DON are suggested to be metabolized to DON prior to absorption and behave in the same way as DON itself, the exposure to DON3G will also result in DON15GlcA levels in urine [4,20]. This is supported by the lack of these forms of DON in urine samples in human biomonitoring studies [3,15,21,22,23]. As such, based on the urinary excreted metabolites in humans, no discrimination can be made between the basic and modified forms of DON. This results in some uncertainties in the model related to the input data of the total dietary intake of DON. We have corrected for the reported co-occurrence of DON and DON3G; however, the estimated dietary intake of total DON did not include possible acetylated forms of DON. Although the contribution of the acetylated forms of DON to the total DON intake may be small [1], it may lead to a discrepancy between the intake and excretion of DON15GlcA in urine and consequently introduce an uncertainty in the established model.

Our study also indicates that DON15GlcA is the major metabolite of DON elimination in humans. DON and DON3GlcA were detected in far less samples and in lower concentrations than DON15GlcA, using analytical methods with comparable detection limits. This was in accordance with various other studies [1,3,14,16], but in contrast to De Ruyck et al., (2020) [24]. De Ruyck et al., (2020) assessed the indirect dietary exposure to mycotoxins via dietary recall and the excretion (of their metabolites) in 24-h pooled urine samples. It was mentioned in this publication that the DON metabolites (glucuronides), DON15GlcA, and DON3GlcA, were analyzed in the urine samples, but these values were not reported in the paper whereas unexpected high levels of acetyl-DON were found in urine. Moreover, Vidal et al., (2018) report no other DON metabolites than DON15GlcA, DON3GlcA, and DOM-1; in some individuals the maximum percentage of recovered DON and metabolites is approximately 95% [3].

Although DON15GlcA is considered DON’s major metabolite, sensitive analytical methods are needed when analyzing this metabolite in urine. Low intake levels of DON and/or combined with highly diluted urine (high individual liquid consumption) require sensitive analytical methods in order to avoid underestimation of the estimated dietary DON intake using reversed dosimetry. By obtaining additional data on the concentrations of DON and DON3GlcA in urine, uncertainties related to the interindividual differences in metabolism (DON15GlcA or DON3GlcA) will be reduced. This, however, also requires lower detection limits for these urinary metabolites. As commercially available standards for DON15GlcA and DON3GlcA are not yet widely available, alternative solutions are to analyze the total excreted amount of DON. Quantitative enzymatic deglucuronidation of the glucuronidated DON metabolites results in free DON, which can subsequently be detected in urine as total DON [13,22,25,26]. However, the quantitative aspect of this deglucuronidation step is still under debate [27].

In our previous publication, a biokinetic model for DON was developed based on a human intervention study [3,9]. The major differences between this study and our previous publication [9], estimating the excretion of DON and metabolites, is that we considered multiple meals (intake moments), missing data (before and after the 24-h time window), more individuals, and we considered an everyday situation rather than an experimental setup. For that human intervention study, quantitative data were available on a single dose administration of DON and the excretion of DON and its metabolites DOM-1 (although this metabolite was found only in small amounts close to the limit of detection in 2 out of 15 individuals), DON3GlcA and DON15GlcA. In the current dataset, sufficient data were available on the intake of DON but limited data on the excretion of DON (only DON15GlcA). As a result, the biokinetic model that was previously developed could not be fitted on the current dataset because that model contains too many parameters that could not be uniquely estimated (because of the lack of DON and DON3GlcA concentrations in urine). Therefore, a statistical model was developed containing a smaller number of parameters. This model only describes the proportion of the intake of DON that is excreted as DON15GlcA (F_abs_excr_) and the parameters of the distribution of the residence time of DON15GlcA, i.e., the distribution of the time duration between the intake and the excretion in urine. The residence time was best described by the model using the gamma distribution.

We estimated that 97.5% of DON15GlcA was excreted after 12.1 h following the intake of DON in an everyday situation. This is similar as described in the human intervention study of Vidal et al., (2018) and Mengelers et al., (2019) after a single administration of DON, where 95% of DON and its metabolites were excreted within 12 h. Of all DON15GlcA, 50% was estimated to be excreted in urine within 4.0 h, which is close to the half-life of DON in human plasma of 2.9–3.6 h as estimated by Fæste et al., (2018) [10].

In our study, the mean fraction of ingested DON that is excreted as DON15GlcA was estimated to be 44% (i.e., a reversed dosimetry factor (RDF) of 2.28). This was also indicated by Mengelers et al., (2019) where a 95% confidence interval of 1.40–7.96 was identified around the RDF of 2.67 for DON15GlcA after DON exposure. This confidence interval was based on the variation in the study population. In our study, the confidence interval for the RDF in the population was estimated to be 0.88–5.91. Although a little smaller in our study, the mean RDF of DON15GlcA and variation therein was estimated to be similar in this everyday situation compared to an experimental setting. This RDF that was derived based on the relation between the dietary exposure of total DON and the urinary excretion of DON15GlcA, can therefore be used to estimate the dietary exposure based on urinary concentrations of DON15GlcA. However, the large variation in the RDFs within the population, reflecting the differences in absorption and excretion of DON between individuals, indicates that it is challenging to estimate the DON exposure based on urinary excretion of DON (and its metabolites) at an individual level. On a population level however, the uncertainty of the RDF will be smaller.

In the human intervention study of Mengelers et al., (2019), a separate RDF of 3 (2.7–3.4) for the combined exposure of a (unknown) mixture of DON and DON3G excreted as urinary DON15GlcA was proposed. This RDF is an average of the individual RDFs of DON and DON3G, whereas we now accounted for the co-occurrence of DON3G in a more realistic manner. The contribution of DON3G in the Norwegian diet was estimated to be 17% (see Materials and Methods). Nonetheless, although both RDFs are quite similar, the two studies, their underlying data, and subsequent derivation of the RDFs differs too much to indicate a preference for one or the other. Actually, it is remarkable that such similar results were obtained whilst using different models, another analytical laboratory, and another intake regime that represented the daily exposure to DON.

We also observed the pattern of DON intake and time to DON15GlcA excretion. First of all, we observed a large variation in the consumption pattern of DON during the whole day. Sampling spot urine in HBM studies with DON is therefore not recommended considering the short half-life of DON15GlcA, i.e., 50% of DON15GlcA is excreted within 4 h. A greater relative variation is generally predicted in spot urine samples, when the elimination half-life is shorter than the interval between exposure [28]. The use of morning urine samples is also not recommended since we found a large variation in the proportion of DON that was consumed during the evening meal relative to the whole day. Even if that variation would have been small, variable urinary excretion voids between evening meal and the first morning urine result in an uncertain exposure estimation based on morning urine. We showed that the median time between the last meal (last dietary intake moment) and the following morning urine is approximately 11.5 h. Simultaneously, we estimated that after 12.1 h, 97.5% of DON15GlcA was excreted, indicating that the DON intake from the evening meal is collected to a very large extent in the urine samples up to the next morning urine. Therefore, we suggest that HBM studies that intent to estimate the daily intake of DON should use urinary samples collected over 24 h (ending with the morning urine sample the next day), rather than spot urine or morning urine samples. This is especially relevant when developing a human biological monitoring guidance value (HBM-GV) as carried out in the HBM4EU project [29].

## 4. Conclusions

This study revealed the challenges when analyzing DON intake and excretion data in an everyday setting. Despite a large variability of the absorbed and excreted fraction between individuals, we showed that a daily DON intake can be estimated based on the 24-h urinary excretion of its main metabolite DON15GlcA, using a mathematical-statistical estimation method that can be used in future HBM studies of DON. This resulted in a population-based RDF reflecting an everyday situation which is in accordance with the results from an experimental setting. This model can be used to assess the external exposure to DON in a population, based only on urinary data of DON15GlcA. For the human biomonitoring of DON, ideally data on all DON metabolites should be used to estimate the DON intake, yet this requires the availability of sensitive analytical methods and reference materials.

## 5. Materials and Methods

### 5.1. Participants

The urine samples used in this study were obtained from the Norwegian biomonitoring study as part of the EU project “European Test and Risk Assessment Strategies for Mixtures” (EuroMix, 633172-2), which was funded by the H2020 programme. The study was approved by the Regional Committees for Medical and Health Research Ethics (REK ID no. 2015/1868) and all the participants provided their written informed consent. All urine voids were collected during a 24-h recording period and two morning urine samples were obtained (t *=* 0 and t *=* 24 h). More information related to the study design, data collection, registration, and processing can be found in Husøy et al., (2019) [17]. In this study, 49 participants (25 males, 24 females) aged 24–64 years were selected from the total of 144 participants of the Norwegian biomonitoring study. These individuals were selected based only on the availability of at least six urine voids and registered grain/bread consumption. As the selected participants in this study are thus not a representative sample of the Norwegian population, we do not consider this a risk assessment of DON in Norway. Of all these participants, dietary intake as well as concentrations of DON and its metabolites were analyzed. As a result of additional exclusion criteria, data from 39 participants were used for the modelling (see Section 5.4).

### 5.2. Urinary Analysis

Urine samples were allowed to thaw at room temperature and aliquots (200 µL) transferred to Eppendorf tubes. Formic acid (50%, 8 µL, Sigma-Aldrich, St. Louis, MO, USA) and ice-cold methanol (200 µL, gradient quality, Romil, Cambridge, UK) were added, and the tubes vortexed for approximately 20 s. The samples were placed at −20 °C for 30 min, followed by centrifugation at 20,000× *g* for 20 min at 4 °C. Finally, the samples were filtered through 0.22 µm Costar Spin-X Nylon centrifuge tube filters (Corning Inc., Corning, NY, USA) at 15,000× *g* for 1 min, and 45-µL-aliquots transferred to fixed-insert HPLC vials to which 5 µL of a solution of U-13C-labelled DON (Romer Labs, Tulln, Austria) in 50% acetonitrile (Romil) was added.

Instrumental analyses were conducted using a Q-Exactive high-resolution mass spectrometer equipped with a HESI-II heated electrospray ionization interface (Thermo Fisher Scientific, Waltham, MA, USA), operated in the negative ionization mode, and a Vanquish Horizon UHPLC pump including autosampler and column oven. Separation was achieved using a Kinetex F5 column (150 × 2.1 mm, 2.6 μm; Phenomenex, Torrance, CA, USA) including a 0.5 μm KrudKatcher Ultra Column In-Line filter (Phenomenex), held at 30 °C with mobile phases A and B of water and acetonitrile (both Optima LC-MS grade, Fisher, Oslo, Norway), respectively, each of which contained 0.2% formic acid. The column was eluted isocratically with 3% B for 1 min, and then using a linear gradient to 15% B over 14 min, followed by a rise to 97% B over 3 min. The column was flushed with 97% B for 2 min, then returned to the starting conditions (1 min), and re-equilibrated for 2 min. The total run-time was 23 min. The mass spectrometer was operated in the full-scan/data-dependent MS2 mode (FS/ddMS2, FS scan range m/z 280–720) and a mass resolution set to 70,000 at m/z 200. Other important instrumental settings included a spray voltage of 2.8 kV, a transfer capillary temperature of 280 °C, a heater temperature of 300 °C, a S-lens RF level of 55, an automatic gain control target of 1 × 106 and a maximum injection time of 200 ms. The settings for ddMS2 included a mass resolution set to 17,500, an automatic gain control target of 1 × 105, a maximum inject time of 100 ms, a loop count of 5 and a quadrupole isolation window of 2 m/z. Quantification of DON was performed based on the [M + formate]^−^ ions (m/z 341.1497) and using internal calibration with reference to the U-13C-labelled internal standard. DON3GlcA and DON15GlcA were quantified selecting the [M−H]^−^ ions (m/z 471.1497) and using external calibration based on matrix-matched calibration standards. While DON for chemical analysis was obtained from Romer Labs, DON-glucuronides were available from earlier work [30]. The retention times for DON, DON3GlcA, and DON15GlcA under above conditions were 5.50 min, 5.90 min and 6.20 min, respectively. The apparent recoveries for DON, DON3GlcA, and DON15GlcA in urine samples are shown in Table 3 and extracted ion chromatograms for DON, DON3GlcA, and DON15GlcA are shown in Appendix A. The limits of detection (LOD) and limits of quantification (LOQ) were calculated from calibration curves from the analyses of all urine samples and were defined as 3× and 10× slope/SE of the slope, respectively. Thus, the LOD/LOQ was determined to 1.0/3.4 ng/mL for DON, 4.6/15 ng/mL for DON3GlcA and 2.0/6.8 ng/mL for DON15GlcA. Actual measured concentrations above the LOD were used for the calculations, while concentrations below the LOD were considered zero and not included in the calculations. This increases the uncertainty in the data, but it was decided to not substitute the data <LOD with e.g., ½ LOD as these were samples with (relatively) high volumes. Table 4 shows the number of urine samples that were analyzed above the LOD.

### 5.3. Dietary Intake

All participants received an open food record, a kitchen scale (brand new and detail to gram), and information on how to record all food and drink items from t = X h on day one to 24 h later on day two. The participants were instructed to eat their habitual diet and to consecutively record all food and drink items and all mixed dishes to the nearest gram (or mL). For mixed dishes, we asked for the name of the dish and a list of single ingredients in addition to the total weight.

Trained research assistants linked all reported food and drink items in the weighted food records to food codes into the food and nutrient calculation system (KBS) at the University of Oslo (UiO). Food intakes in grams per day were multiplied with DON occurrence in foods in a deterministic approach. A database was constructed for DON occurrence using two approaches: (i) the lowest annual mean DON concentrations measured in 111 samples of Norwegian milled wheat flour, sieved wheat flour, wheat bran, and oat flakes during the years 2008 to 2011, and (ii) the highest annual mean DON concentrations from the same sampling period. Samples below the limit of detection (LOD) were set to half the LOD. Less than 10% of the samples in each flour category had values below the LOD and had little impact on the total mean concentrations of DON [18,31]. As DON content was measured in flour, not in actual foods, recipes were used to estimate the DON concentration (µg per 100 g) in different types of bread, rolls, and other bakery wares and likewise for products including wheat bran and oat flakes. For breakfast cereals other than oat flakes, rye bread, and pasta, we used the mean medium bound DON values from the European occurrence data reported in the EFSA risk assessment of DON [1].

A range of DON content for every meal was estimated by multiplying the DON concentrations (based on the lowest and highest annual mean concentrations) in the food products by the consumed amounts of the respective food products. For each participant, the daily DON exposure was divided by body weight and expressed as µg DON/kg bw/day. The intake of modified forms of DON were not included in these data as occurrence data were not available. Therefore, we corrected for the additional dietary exposure of DON3G with a ratio 0.17, according to the values presented by EFSA for the northern European countries, while also correcting for the molecular weight of DON3G [1]. This resulted in an additional DON intake from DON3G of 11%. Because of the different molecular weights of DON and DON15GlcA, we defined our model in terms of moles and not grams.

### 5.4. Statisical Model and Data Eligibility

In our previous publication [9], data were available on the intake of a single dose of DON and the excretion of DON and its metabolites DOM-1, DON3GlcA, and DON15GlcA. In the current dataset, only sufficient data were available on the intake of DON and the excretion of DON15GlcA. For the other substances analyzed in urine (DON and DON3GlcA), most excretion values were below the detection limit and could therefore not be properly quantified (Table 4). Therefore, a mathematical statistical approach was taken to describe the data with a smaller number of parameters that could be identified, rather than the biokinetic model (see Appendix B). Because of the nature of the current data, this statistical model describes the proportion of the intake of DON that is excreted as DON15GlcA (f_abs_excr_) and the distribution of the residence time, i.e., the time between the DON intake and the excretion of DON15GlcA in urine.

The parameters in the descriptive statistical model that were estimated are the f_abs_excr_-parameter and the parameters of the residence time (average, μ, and variation, σ). This f_abs_excr_ is the product of the absorbed proportion of DON into the body and the excreted fraction of DON15GlcA into urine. In our previous publication we found that the population cannot be assumed to be homogenous with respect to the absorption of DON [9]. Therefore, we also addressed the heterogeneity in the population by assuming the f_abs_excr_-parameter to be random. The data did not allow both parts of the model to be random (data not shown). Therefore, we assumed the residence time distribution as being fixed for all individuals, and only the f_abs_excr_ as being different. This results in a so-called mixed effects statistical model, of which the details are described in Appendix C. The DON intake and DON15GlcA excretion data were used to fit the model and to estimate the model parameters.

Various statistical distributions were used to describe the residence time of DON15GlcA over time for all individuals, namely a gamma distribution, a log-normal distribution and an exponential distribution. These distributions must share two constraints: non-negative time values and a skewed distribution (where the mean value is larger than median value). The difference between these distributions mainly applies to the shape of the tails of the distribution. The gamma and log-normal statistical distributions are bivariate distributions and contain two parameters: μ, which relates to the average, and σ, which relates to the variation. The exponential distribution contains only one parameter, that defines both average and variation.

The data of both males and females were grouped together in the model to estimate the residence time and f_abs_excr_, similar to our previously developed biokinetic model on DON [9].

Contrary to our previous publication, the current data were obtained from an everyday setting instead of an experimental setting. That means that there can be zero (although unlikely) or multiple intake time points during the one-day time-window. Moreover, the 1st morning excretion amount relates to a previous and unknown intake amount outside our 24-h time window and in addition, the last evening intake may result in an unknown excretion amount after the last urine void (morning urine) the next day. In statistical theory, these unknown values are known as missing values. To address this issue, we only included the data from individuals with at least two positive excretion data points, with the starting excretion time point being after at least 1 h after the 1st morning intake. This resulted in the exclusion of the data from five individuals. Moreover, to prevent the fitting procedure to estimate very large mean residence time duration values due to missing excretion values, we assumed (based on the results of our previous study) the last intake amount would not result in a measurable excreted amount after more than 20 h (Appendix D). In addition, we excluded data from individuals with a total urinary excretion of DON15GlcA over 24 h was substantially greater than the dietary intake of total DON over 24 h, while correcting for the molecular weight (with a factor 1.1, see Appendix D) and accounting for the uncertainty in the intake data by accepting a maximal overexcretion of 27%. This value was estimated by firstly calculating the individual average dietary intake based on the upper and lower intake estimations. The average of all upper intake estimations was divided by the individual average dietary intake, resulting in a mean value of all the ratios of 1.27. This exclusion criteria resulted in the exclusion of another five individuals. In total, the data of 39 individuals were included in the modelling (see Appendix D).

Because of the different molecular weights of DON and DON15GlcA, we defined our model in terms of (micro)moles and not (micro)grams. After we applied the exclusion criteria as described previously, we applied the nlme-procedure (version 3.1) in R (version 4.0.2) to fit the model on the data.

To compare all model fits, we applied the Akaike Information Criterium (AIC). The AIC-value quantifies the model fit, taking into account the number of parameters included in the model [32]. The preferred statistical model to describe the excretion of DON15GlcA over time is the one with the lowest AIC values.

### 5.5. Validation Steps

Several assumptions that were made were validated using post-analysis methods. We assessed whether the assumption of a normally distributed random effect variable and normally distributed error terms were valid by drawing so-called QQ-plots of the calculated log-transformed f_abs_excr_ values and the residuals, respectively. Moreover, we assessed whether the assumption of independently distributed error terms was valid by regressing the residuals to the absolute excretion values and to the time passed since intake (see Appendix E).

### 5.6. Reversed Dosimetry Factor

Using the urinary excretion levels of DON15GlcA, the intake of total DON can be assessed for exposure and/or risk assessment purposes. The cumulative excreted amount of DON15GlcA in moles can be multiplied by a reversed dosimetry factor (RDF) to obtain the equivalent amount of total DON intake, in moles. Based on the results of this study, the inverse of the f_abs_excr_ was used to derive a RDF to estimate future total DON intakes (Appendix F). The f_abs_excr_ was estimated by the random part of the mixed effects model (see Appendix C). The 95% uncertainty around the population mean RDF was calculated by dividing the RDF by the square of the uncertainty (lower boundary) and multiplying the RDF by the square of the uncertainty (upper boundary).

## Figures and Tables

**Table 1:** Concentrations of DON15GlcA in urine of all the individuals in this study.

*n*	Total 49	Males 25	Females 24
Average amount DON15GlcA (µg)/24 h (min–max)	26.1 (0–91.3)	33.4 (0–91.3)	18.6 (0–57.2)
Average concentration DON15GlcA (µg/mL)/24 h (min–max)	0.02 (0–0.11)	0.02 (0–0.11)	0.01(0–0.04)
Average urine volume in 24 h (mL) (min–max)	2107 (770–4190)	2148 (770–4190)	2064 (950–3845)
Average urine flow in 24 h (mL/kg bw) (min–max)	29.1 (9.10–62.0)	26.6 (9.10–55.1)	31.8 (13.6–62.0)

**Table 2:** The modelled residence time and excreted fraction after fitting the data in the model using the different statistical distributions based on 39 individuals in this study.

		Gamma	Log-Normal	Exponential
Residence time	Mean	4.70	4.15	2.89
	Standard deviation	2.99	2.43	2.89
	Median	3.97	3.58	2.00
	97.5%	12.1	10.4	10.7
AIC ^1^		685	739	767
F_abs_excr_	Population mean	0.44	0.47	0.44
	Relative uncertainty ^2^	0.10	0.11	0.11
	Relative heterogeneity ^3^	0.61	0.62	0.62
RDF	Population mean (95% confidence interval)	2.28 (1.88–2.76)	2.14 (1.74–2.64)	2.27 (1.84–2.80)
	Population heterogeneity (95% confidence interval)	2.28 (0.88–5.91)	2.14 (0.82–5.62)	2.27 (0.86–5.96)

^1^ Akaike Information Criterium. ^2^ The relative uncertainty around the F_abs_excr_ reflects the uncertainty of the F_abs_excr_ as estimated in the model, see Materials and Methods. ^3^ The relative heterogeneity of the F_abs_excr_ reflects the variation of the F_abs_excr_ in the population, see Materials and Methods.

**Table 3:** Apparent recovery for DON, DON3GlcA, and DON15GlcA during the period the urinary analyses were conducted.

	Theoretical Concentration (ng/mL)	Mean Apparent Recovery (%)	Standard Deviation (%, *n* = 15)
DON	20.1	99	8.7
DON3GlcA	19.8	93	17
DON15GlcA	20.1	86	21

**Table 4:** Number of urine samples that were analyzed above the LOD for DON, DON3GlcA, and DON15GlcA.

>LOD	*n*	%
DON	70/436	16
DON3GlcA	76/436	17
DON15GlcA	304/436	70

**Figure A1:**
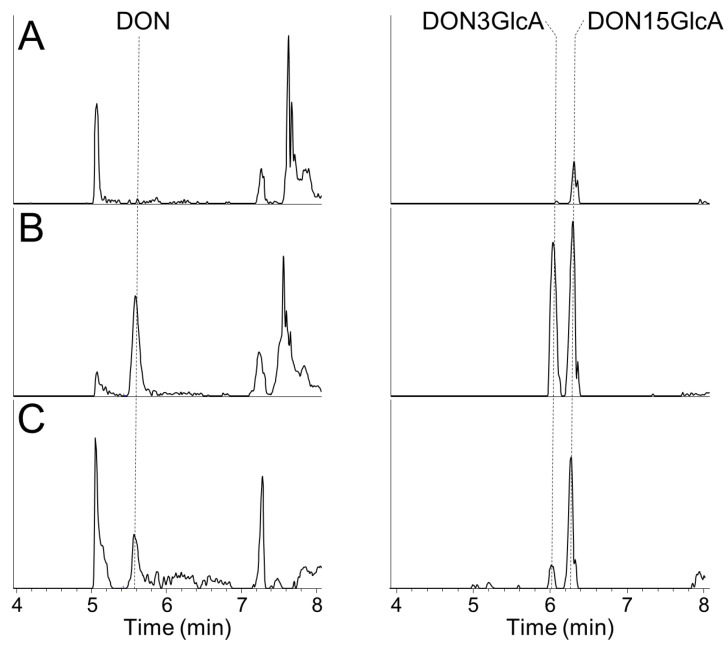
Extracted ion chromatograms (±3 ppm) from full-scan LC–HRMS for the formate adduct of DON (left) and the deprotonated molecules of DON–glucuronides (right). (**A**) Negative control urine sample (containing traces of DON15GlcA), (**B**) urine sample fortified with 20 ng/mL each of DON and DON–glucuronides, (**C**) urine sample containing 10 ng/mL DON and 13 ng/mL DON15GlcA (DON3GlcA was below the calculated LOD). Chromatograms are on a fixed scale with maximum peak intensities normalized to 2.5 × 106 for DON-formate and 1.5 × 104 for negatively charged DON–glucuronides.

**Figure A2:**
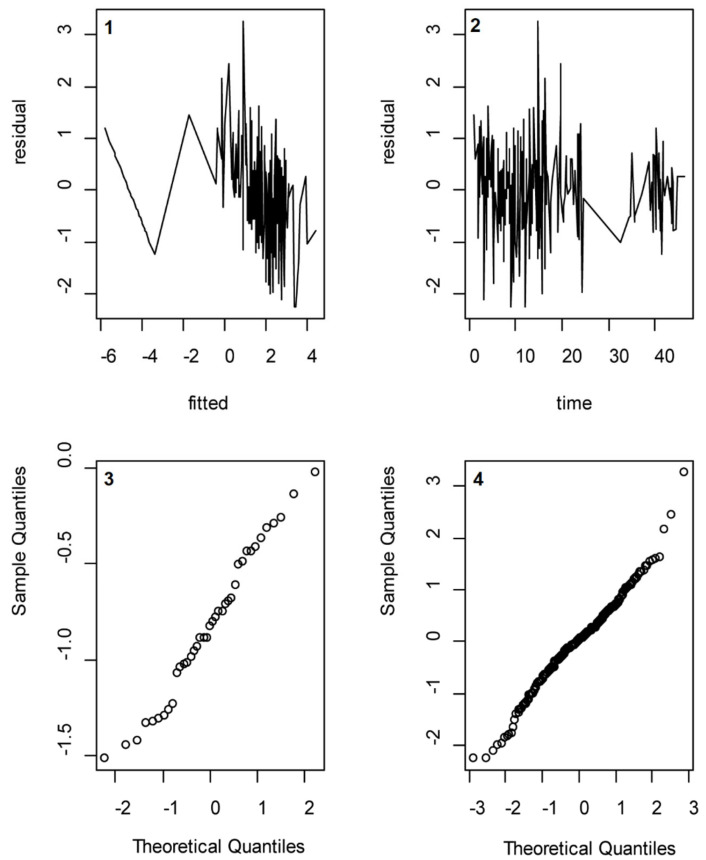
Validation of the assumptions made. Graph shows the residuals plotted against the fitted values, the second graph shows the residuals plotted against time, the third graph shows the QQ-plot of the calculated individual f_abs_excr_ values, and the fourth graph shows the QQ-plot of the residuals.

**Figure A3:**
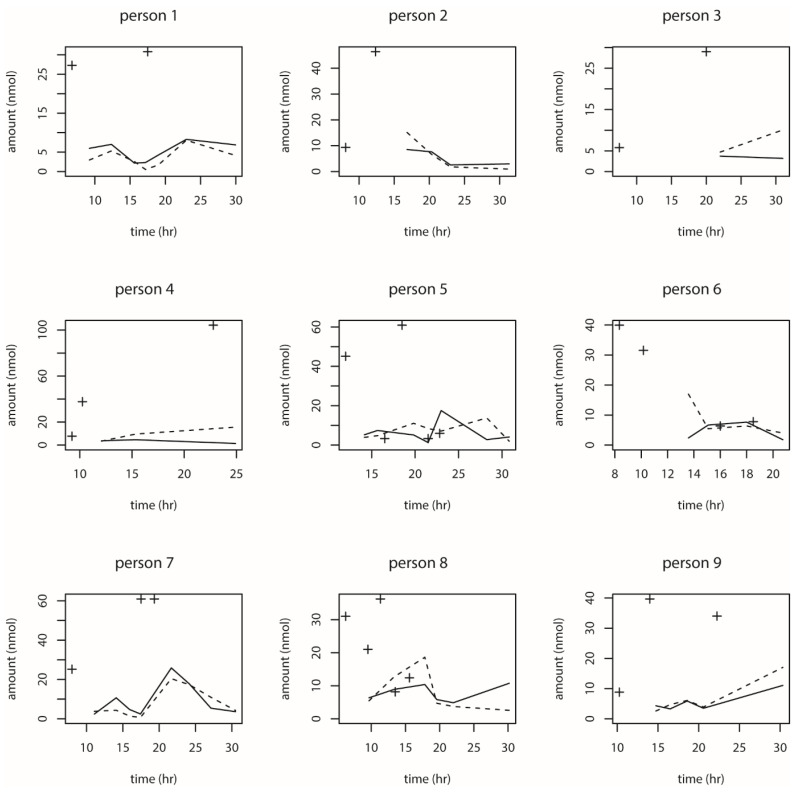

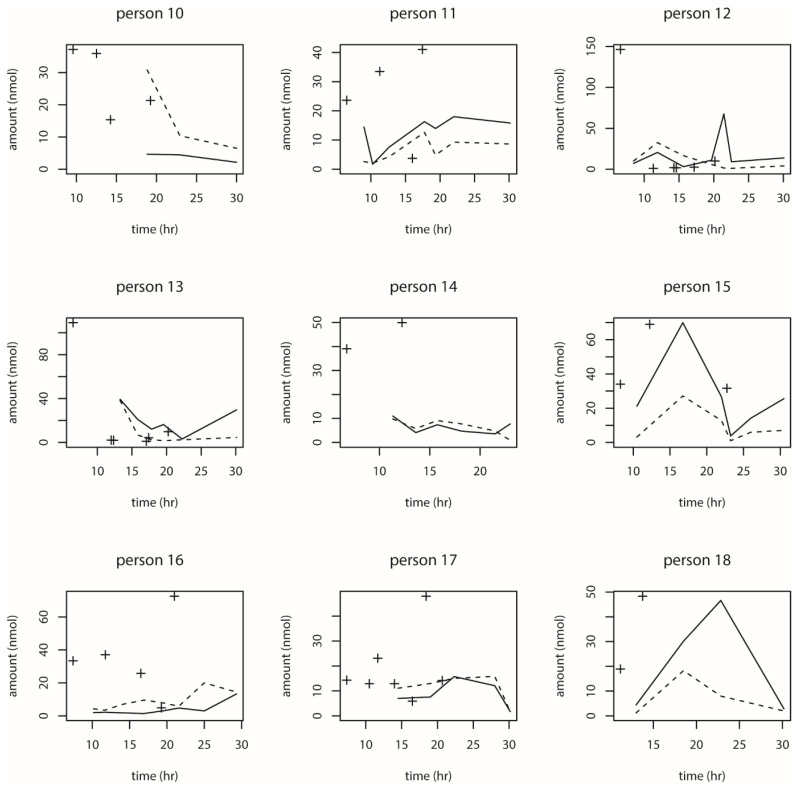

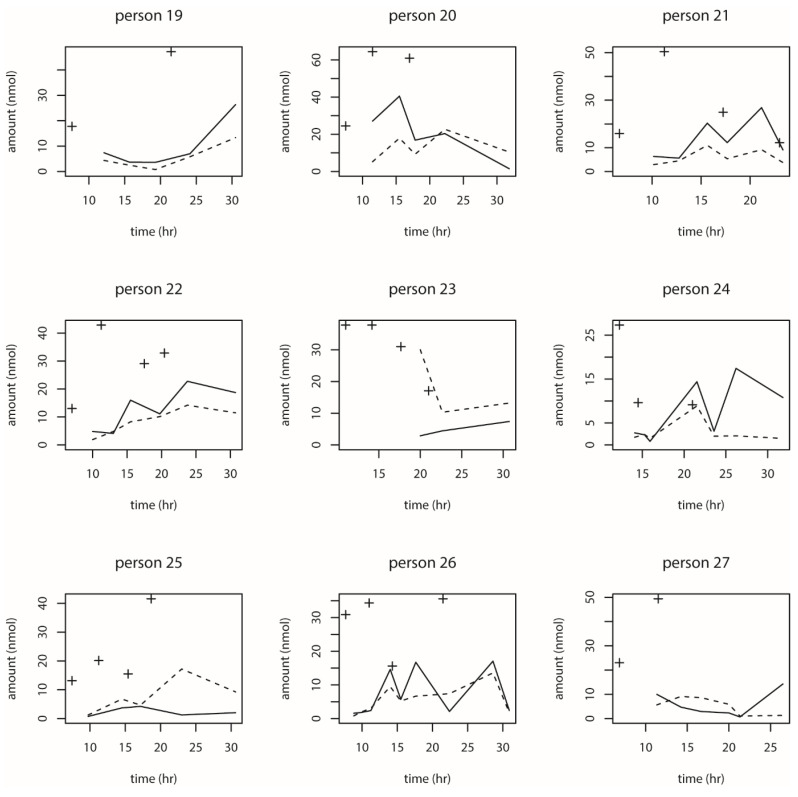

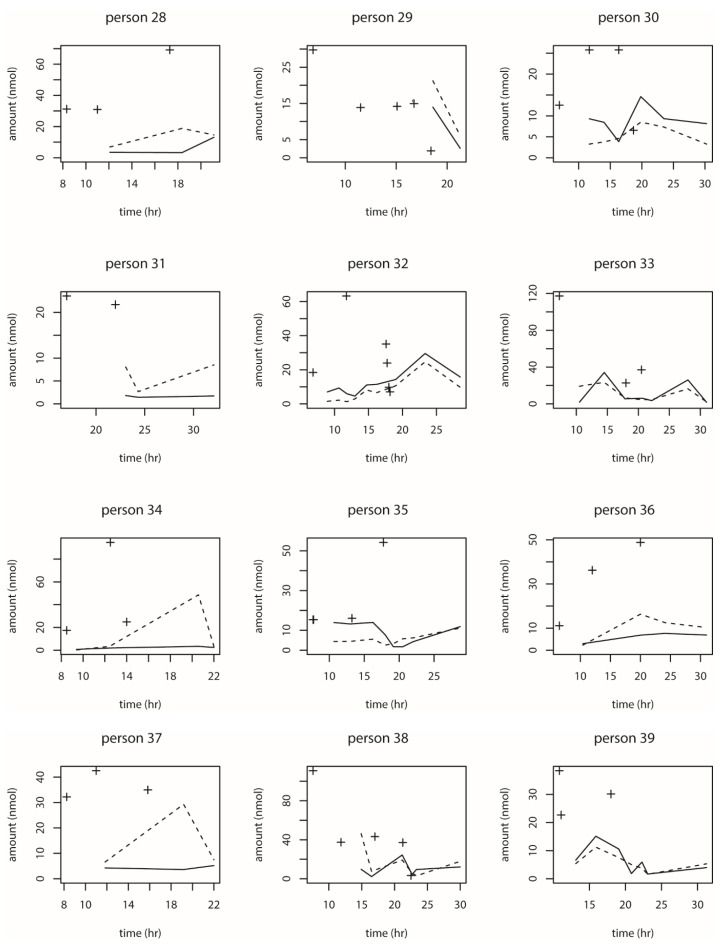
Dietary DON intake and urinary DON15GlcA excretion for every individual in this study. Solid lines represent the excreted data as analyzed in urine, dashed lines represent the excreted data as fitted by the model. + indicates the dietary intake. Data is shown as nanomoles (nmol) against time in hours during the day (hr).

## Appendix B

The mathematical formula that that describes the excretion as a function of the intake and the to be estimated parameters are as follows:

(A1) $$CumN_{excr}\left(t\right)=\sum_{i}f_{abs\_excr}N_{inta}\left(t_{i}\right)F\left(t-t_{i};\mu ,\sigma \right)$$

with:

CumNexcr (t) cumulative excretion amount at time t;
ti timepoints of the intake, with t_i_ < t;
i index on the intake;
Ninta(ti) intake amount at time t_i;_
F (t – ti;μ,σ) the cumulative distribution function (e.g., gamma, lognormal, or exponential distribution) of the residence time with the to be estimated model parameters μ (average) and σ (variation);
fabs_excr the to be estimated model parameter that describes the proportion of the intake of DON that is excreted as DON15GlcA.

The excretion amount at time point t_j_ can then be described by:

(A2) $$N_{\mathrm{excr}}\;(t_{j})={\mathrm{CumN}}_{\mathrm{excr}}\;(t_{j})-\mathrm{Cum}\;N_{\mathrm{excr}}\;(t_{j-1})=\sum_{i}f_{abs\_excr}N_{inta}\left(t_{i}\right)\left(F\left(t_{j}-t_{i};\mu ,\sigma \right)-F\left(t_{j-1}-t_{i};\mu ,\sigma \right)\right)$$

with:

j index of the urine voids

These formulas show that the characteristics of the distribution function F affect the contribution of the intake at the excretion time points; the bigger μ and σ are, the longer the contribution is maintained over time. The formulas therefore also show that as a result of the study design, the first excretion relates to an unknown intake and the latest intake relates to an unknown excretion.

## Appendix C

Based on the presented mathematical formula that describes the excretion as a function of the intake model (Appendix B) and the assumed heterogeneity of the population, a mixed-effects model was derived. This statistical model is based on a regression model with one random parameter. The regression model describes the outcome variable as a function of the to be estimated model parameters (f_abs_excr_, μ and σ). The random part describes the to be estimated distribution of the f_abs_excr_ parameter as a stochastic variable, with its distributional characteristics to be estimated. The parameters μ and σ are assumed to be fixed.

### Appendix C.1

The regression model describes the outcome variable as a non-linear function of the to be estimated parameters, i.e., f_abs_excr_, μ and σ (see Appendix B).

(A3) $$\log\;N_{\mathrm{excr},k}\;(t_{j,k})\;\sim \;\log(\sum_{i}f_{abs_{excr},k}N_{inta,k}\left(t_{i,k}\right)\;\left(F\left(t_{j,k}-t_{i,k};\mu ,\sigma \right)-F\left(t_{j-1,k}-t_{i,k};\mu ,\sigma \right)\right)$$

with:

fabs_excr,k f_abs_excr_ for individual k;
Ninta,k (ti,k) intake amount of individual k on time t_i,k_;
Nexcr,k (tj,k) excretion amount of individual k on time t_j,k_;
ti,k intake time points of individual k;
tj,k excretion time points of individual k, t_j-1,k_ describes the previous excretion time point of individual k (i.e., the excretion between two excretion time points);
F the cumulative distribution function of the residence time (e.g., gamma, lognormal, or exponential distribution).

The log-transformation was applied to adjust for the skewness of the distribution of the excretion, see also the section on model validation.

### Appendix C.2

The random part of this model describes the f_abs_excr_ parameter as being log-normally distributed over the population.

log (f_abs_excr,k_) ~ N(μ_fabs_excr_, σ_fabs_excr_) (A4)

with:

μfabs_excr, σfabs_excr average and variation of log (f_abs_excr_) in the population

Summarizing, the to be estimated parameters are μ_fabs_excr_, σ_fabs_excr_ (that describe the distribution of f_abs_excr_ in the population) and μ, σ (that define the distribution of the time between intake and excretion).

## Appendix D

The data in this study were obtained from participants in an everyday situation, in contrast to the experimental setting described in Mengelers et al., (2019). This practical situation resulted in the application of additional modeling steps.

### Dealing with Missing Values

The obtained excretion from the 1st morning urine relates to a previous, unknown intake outside our 24-h time window. In addition, the last intake of the day may result in an unmeasured excretion after the last urine void (2nd morning urine), i.e., after our 24-h time window. These unknown values are known as missing values in statistical theory. To deal with the missing intake values we only included excretion time points (voids taken) after at least 1 h after the 1st morning intake.

To deal with possible missing excretion values we generated two additional excretion data points after the last meal (intake moment). The 1st additional excretion data point is set 20 h after the last meal, and has a missing, non-zero excretion amount value. The 2nd additional data point is set 30 h after the last intake value, and has a missing excretion value of zero. This prevents the fitting procedure from selecting very large mean residence time duration values due to missing excretion values, see Figure 3. The density function of the statistical model for the excretion of DON15GlcA in a fictional individual, assuming the residence time parameters of the statistical model using the gamma distribution for the residence time. The amount of DON15GlcA excreted in urine is reflected by the area under the curve between two excretion time points (vertical black lines at t = 9, 16, 23, and 31 h). The modeled additional excreted amounts of DON15GlcA are generated at t = 38 and t = 48 h (dashed lines). Intake time points were assumed at t = 8 and t = 18 h (dotted lines).

Missing intake values might also result in (cumulative) excretion values being too large compared to the (cumulative) intake values. Therefore, we compared the measured intake and excretion values (with the excretion values starting 1 h after the 1st intake value, see previous section), and excluded individuals if the ratio of the measured excretion amount to the measured intake amount exceeded an upper limit value. The upper limit value is calculated as the product of two terms. The 1st term relates to a systematic error. The measured intake relates to DON whereas the measured excretion relates to all products from intakes of both DON and DON3G, the latter not being measured. Thus, the amount of intake of DON3G given the intake of DON was estimated using the ratio 0.17 (85:15; in terms of weight). The formulas applied are:

(A5) $$\frac{w_{2}*n_{2}}{w_{1}*n_{1}}=\frac{15}{85}\;\to \;n_{2}=\frac{w_{1}}{w_{2}}*\frac{15}{85}*n_{1},\;n_{2}+n_{1}=\left(1+\frac{w_{1}}{w_{2}}*\frac{15}{85}\right)*n_{1}=1.11*\;n_{1}$$

with:

index i = 1: DON, i = 2: DON3G;
ni number (moles);
wi molar weight; w_1_ = 296.3, w_2_ = 458.5.

The resulting value in molar terms is 1.11, meaning that an intake of 1 micromole DON corresponds with 1.11 micromole DON and DON3G combined. The 2nd term relates to an uncertainty error being related to the intake values. We calculated the uncertainty error from the uncertainty bounds of the intake values. We first calculated the ratios of the reported upper boundary value to the reported mean value for all intakes, and then calculated the mean value over all ratios, with resulting value 1.27. So, individuals with total excretion values being larger than 1.11 × 1.27 = 1.41 times the total intake value were excluded from the analysis.

The exclusion criteria that resulted in the exclusion of ten individuals were: at least 1 non-missing excretion value (3 participants excluded), at least 1 non-missing intake value and total excretion below upper limit (5 participants excluded), at least 2 excretion values (2 participants excluded). This resulted in 39 out of the 49 individuals being selected for the further analyses.

## Appendix E

Figure A2 shows the validation steps of the statistical model using a gamma distribution. The first graph (1) shows the residuals plotted against the fitted values, and the second graph (2) shows the residuals plotted against time. Note that the fitted values are on the log-scale, because of the log-transformation of the data before fitting the model. The graphs show there is no systematic effect of size of fitted values or of time on the residuals. The third graph (3) shows the QQ-plot of the calculated individual f_abs_excr_ values, and the fourth graph (4) shows the QQ-plot of the residuals. These graphs show that the assumptions of normally distributed f_abs_excr_ values and normally distributed error terms are both valid.

## Appendix F

The reversed dosimetry factor (RDF) is the inverse of the f_abs_excr_, the fraction of the DON intake excreted as DON15GlcA in urine. As shown in the previously presented formula, the f_abs_excr_ describes the relation between the intake and (cumulative) excretion when we assume one meal and an infinite t:

(A6) $$CumN_{excr}\left(t_{\infty }\right)=\underset{t\to \infty }{\lim}CumN_{excr}\left(t\right)=f_{abs\_excr}N_{inta}\left(t_{1}\right)$$

Thus:

(A7) $$RDF=\frac{N_{inta}\left(t_{1}\right)}{CumN_{excr}\left(t_{\infty }\right)}=\frac{1}{f_{abs\_excr}}$$

This indicates that the RDF has a similar structure as the f_abs_excr_, i.e., with a population average and an individual variation. In case of the gamma-distributed residence time, the population mean value of the RDF is 2.28 with 95% confidence bounds 2.28/1.10^2^ = 1.88 and 2.28 × 1.10^2^ = 2.76 respectively. The individual variation of the RDF is 2.28 with 95% confidence bounds 2.28/1.61^2^ = 0.88 and 2.28 × 1.61^2^ = 5.9 respectively.

## Appendix G

Below, Figure A3 shows the individual excreted DON15GlcA amounts as analyzed as well as estimated using the model with a gamma distribution of the residence time.
